# Growth from infancy to adulthood and associations with cardiometabolic health in individuals born extremely preterm

**DOI:** 10.1016/j.lanwpc.2023.100717

**Published:** 2023-02-27

**Authors:** Jeanie L.Y. Cheong, Joy E. Olsen, Tayla Konstan, Rheanna M. Mainzer, Leah M. Hickey, Alicia J. Spittle, John D. Wark, Michael M. Cheung, Suzanne M. Garland, Julianne Duff, Marissa Clark, Penelope Stevens, Lex W. Doyle, Victorian Infant Collaborative Study Group, Peter Anderson, Rosemarie Boland, Alice Burnett, Margaret Charlton, Marissa Clark, Noni Davis, Lex Doyle, Julianne Duff, Leah Hickey, Emily Johnston, Elisha Josev, Katherine Lee, Rheanna Mainzer, Marion McDonald, Bronwyn Novella, Joy Olsen, Gillian Opie, Lauren Pigdon, Gehan Roberts, Alicia Spittle, Penelope Stevens, Alice Stewart, Anne-Marie Turner, Tania Woods

**Affiliations:** a Clinical Sciences, Murdoch Children's Research Institute, Melbourne, Australia; b Newborn Research, Royal Women's Hospital, Parkville, Victoria, Australia; c Department of Obstetrics and Gynaecology, University of Melbourne, Melbourne, Australia; d Department of Paediatrics, University of Melbourne, Melbourne, Australia; e Clinical Epidemiology and Biostatistics Unit, Murdoch Children's Research Institute, Melbourne, Australia; f Neonatal Medicine, Royal Children's Hospital, Melbourne, Australia; g Department of Physiotherapy, University of Melbourne, Melbourne, Australia; h Department of Medicine, University of Melbourne, Melbourne, Australia; i Bone and Mineral Medicine, Department of Diabetes and Endocrinology, Royal Melbourne Hospital, Melbourne, Australia; j Department of Cardiology, Royal Children's Hospital, Melbourne, Australia; k Heart Research, Murdoch Children's Research Institute, Melbourne, Australia; l Centre for Women's Infectious Diseases Research, Royal Women's Hospital, Parkville, Australia; m Infection and Immunity, Murdoch Children's Research Institute, Parkville, Australia; n Neonatal Services, Mercy Hospital for Women, Melbourne, Australia; o Department of Neonatology, Monash Medical Centre, Melbourne, Australia

**Keywords:** Extremely preterm, Growth, Weight, Height, Body mass index, Cardiometabolic health

## Abstract

**Background:**

Few studies have tracked growth in children born extremely preterm (EP, <28 weeks’ gestation) beyond late adolescence. The relationships between growth parameters (including weight and BMI) through childhood and adolescence with later cardiometabolic health, are unclear in those born EP. We aimed to (i) compare growth from 2 to 25 years between EP and controls; and in the EP group (ii) determine the associations of growth parameters with cardiometabolic health.

**Methods:**

Prospective state-wide cohort of all EP livebirths in Victoria, Australia, in 1991–1992 and contemporaneous term-born controls. Z-scores for weight (z-weight), height (z-height) and BMI (z-BMI) at 2, 5, 8, 18 and 25 years, and cardiometabolic health at 25 years (body composition, glucose tolerance, lipid profiles, blood pressure, exercise capacity) were measured. Growth trajectories were compared between groups using mixed models. The relationships between z-BMI changes/year, and being overweight at different ages, with cardiometabolic health were explored using linear regression.

**Findings:**

Z-weight and z-BMI were lower in EP than controls, but the gap decreased with age due to a more rapid rate of rise in z-weight and a decrease in z-height in the EP group compared with controls. Greater increases in z-BMI/year in the EP group were associated with poorer cardiometabolic health [coefficient (95% CI) per 0.1 z-BMI increase/year: visceral fat volume (cm^3^) 217.8 (160.9, 274.7), triglycerides (mmol/L) 0.45 (0.20, 0.71), systolic blood pressure (mmHg) 8.9 (5.8, 12.0), and exercise capacity (BEEP test maximum level −1.2 (−1.7, −0.7)), all p < 0.001]. The association between being overweight with poorer cardiometabolic health strengthened with age.

**Interpretation:**

The catch-up in weight and BMI by young adulthood in survivors born EP may not be desirable as it is associated with poorer cardiometabolic health. The associations of being overweight from mid-childhood with poorer cardiometabolic health may provide a window for intervention.

**Funding:**

10.13039/501100000925National Health and Medical Research Council of Australia.

**Research in context**

**Evidence before this study**

Individuals born extremely preterm have poorer growth than those born full-term. Catch-up weight is reported in childhood but whether this continues into older ages is unclear. The associations between growth and later cardiometabolic health in extremely preterm individuals is not well described.

**Added value of this study**

Catch-up in weight and BMI, but not height, continues through childhood into young adulthood in survivors born extremely preterm. Being overweight from mid-childhood (8 years), but not younger ages, is associated with poorer cardiometabolic health in adulthood.

**Implications of all the available evidence**

As greater catch-up in weight is associated with poorer cardiometabolic health, there is a need to determine the optimal catch-up growth for those born extremely preterm. There may be a window of opportunity for intervention in early childhood, as associations with poorer cardiometabolic health become stronger from mid-childhood, but not at younger ages.

## Introduction

Poor growth in infancy and childhood is well documented in cohorts born extremely preterm (EP, <28 weeks’ gestation).^1^, ^2^, ^3^ Several studies of EP cohorts have reported positive associations between growth and neurodevelopment,^4^^,^^5^ thus leading to an emphasis on maximising early nutrition, from the first days to months after discharge, to improve growth and, consequently, neurodevelopment.^6^

Catch-up growth in childhood, however, is not without concerns as there may be increased risk for later cardiometabolic morbidity.^7^ Obesity in childhood is associated with later risk for cardiometabolic disease.^8^ This risk may be greater in those born EP, where altered early environments antenatally and in early postnatal life may further negatively influence their risk of non-communicable disease in later life, including cardiometabolic disease.^9^

Advances in maternal and neonatal care since the early 1990s has seen a dramatic rise in survival,^10^ with large numbers of EP infants now reaching early adulthood. Although there are reports of growth trajectories into adult life in preterm cohorts, many of the earlier reports have been on cohorts selected by birth weight, rather than gestational age.^11^ Thus, the research findings may be biased by inclusion of more mature but growth restricted individuals. Further, there is limited research reporting growth trends of EP cohorts that extend beyond late adolescence. Moreover, the relationships between being overweight at different ages in childhood and adolescence in EP cohorts and later cardiometabolic health are not well described. To address these research gaps, we undertook a prospective longitudinal cohort study focussed on growth trajectories and their relationships with cardiometabolic health in young adulthood. Specifically, we aimed to compare between EP and controls: (i) z-scores for weight (z-weight), height (z-height), and BMI (z-BMI) at ages 2, 5, 8, 18, and 25 years, and (ii) growth, i.e., z-weight, z-height, and z-BMI changes between 2 and 25 years. We also aimed to explore the associations between growth velocity (z-BMI changes/year between 2 and 25 years), and being overweight at ages 2, 5, 8, 18, and 25 years, with cardiometabolic health at 25 years in the EP group.

## Methods

Participants were all survivors born EP (n = 225) in the state of Victoria, Australia, between 1st January 1991 and 31st December 1992 who were recruited at birth as part of the Victorian Infant Collaborative Study. Contemporaneous healthy term-born (37–42 weeks' gestational age) and normal birthweight (>2499 g) controls (n = 253) were also recruited at birth, and were matched for sex, expected date of birth, the mother's health insurance status (private or public, as a proxy for social class), and the primary language spoken in her country of birth (English or other).

The study was approved by the Human Research Ethics Committees at The Royal Women's Hospital, Mercy Hospital for Women and Monash Health in Melbourne, and all participants provided written informed consent. The present data analysis was conducted from 14th April 2022 to 29th June 2022. The reporting to this study followed the Strengthening the Reporting of Observational Studies in Epidemiology (STROBE) reporting guideline.

Perinatal data were collected prospectively. Participants were seen for follow-up at 2, 5, 8, 18, and 25 years, corrected for prematurity, for neurodevelopmental and health assessments, by assessors who were blinded to group, clinical history and previous assessments.^12^ Body mass index (BMI) was calculated as weight (kg)/height (m)^2^. Z-weight, z-height and z-BMI were calculated using the UK WHO Preterm growth charts at birth and two years,^13^ and the British Growth Reference (based on the 1990 UK version) at 5, 8, 18 and 25 years.^14^ At 25 years, we used the maximum reference value on the growth chart of 23 years for participants who were >23 years’ corrected age when assessed as there are no appropriate height and weight reference data for 25-year-olds. Participants were categorised as overweight according to age and sex specific cut-offs for BMI.^15^

At 25 years, participants attended a full day assessment, the protocol for which has been published.^12^ All measures were conducted by experienced assessors who were blinded to birth group, clinical history, and prior assessments.

Height (m) and weight (kg) were measured using digital measuring station Seca 284 (Hamburg, Germany). Visceral fat volume was estimated using dual-energy x-ray absorptiometry and Hologic Horizon APEX System Software version 5.5.3 (Hologic Horizon A, Hologic Inc, MA). Participants fasted overnight for at least 8 h, prior to blood samples being taken for insulin, glucose and lipid assays. The Homeostasis Model Assessment Insulin Resistance Index (HOMA-IR) was calculated with the formula: fasting serum insulin (mU/L)∗ fasting plasma glucose concentration (mmol/L)/22.5.^16^ Cholesterol, high-density lipoprotein (HDL), low-density lipoprotein (LDL) and triglyceride levels were also measured.

Ambulatory blood pressure was measured over a 24-h period using an Oscar 2 ambulatory BP monitor (SunTech Medical Inc, Morrisville, NC). Systolic, diastolic, and mean arterial pressure readings were taken every 30 min when expected to be awake and every 60 min when expected to be asleep. The values reported are the averaged readings over the 24 h.^17^

Functional fitness and exercise tolerance were measured using the Six-Minute Walk Test^18^ and the Beep Test.^19^ The Six-Minute Walk Test assesses the total distance walked on a flat surface in 6 min. It is a widely used assessment and is predictive of cardiac health outcomes in adults. For the Beep Test, a commonly used assessment of aerobic fitness, participants were required to run between two 1s 20 m apart, at increasing speeds. The maximum level was recorded when participants were not able to keep up with the running speed specified by the beep recording.

### Statistical analyses

Data were analysed using Stata 17 (StataCorp, Texas, USA). Mean differences in growth parameters between EP and controls were estimated at each time-point using linear regression, fitted using generalised estimating equations with an exchangeable correlation structure to account for clustering of multiple births. Trajectories of growth from 2 to 25 years for z-weight, z-height and z-BMI were compared between groups using mixed effects models, with a fixed effect for group, and age (in years); and a random effect for individual to allow for repeated measures within individuals. We also added a random effect for age to allow for random slopes, after performing likelihood ratio tests. We added an interaction between group and age to test whether the EP and controls differed in slope. The effect of sex was examined by adding this to the model as an additional fixed covariate, and an interaction between sex and age. Mixed effects models were fitted using restricted maximum likelihood and an unstructured covariance matrix between random effects.

The relationships between z-BMI change per year between 2 and 25 years (estimated for each individual using linear regression), and being overweight at 2, 5, 8, 18, and 25 years, with continuous variables associated with cardiometabolic health (visceral fat volume, fasting insulin, glucose, and lipid levels, blood pressure and fitness measures) at 25 years in the EP group were assessed using separate univariable linear regression models for each predictor and outcome. All models were fitted using generalised estimating equations with an exchangeable correlation structure to account for clustering of multiple births. To allow for the associations of being overweight at different ages with each cardiometabolic health outcome variable at 25 years to be compared relative to one another, we expressed the associations as % differences relative to the mean of each outcome variable for each age. We do not adjust for multiple comparisons or make conclusions based on p-value thresholds. Instead, we focus on the overall strength of evidence of the relationships between variables assessed, rather than each relationship in isolation.

### Role of funding source

The funder had no role in the study design, the collection, analysis, interpretation of data, the writing of the report; and in the decision to submit the paper for publication. Authors have not been paid to write this article by a pharmaceutical company or other agency. Authors were not precluded from accessing data in the study, and the corresponding author accepts responsibility to submit for publication.

## Results

### Participants

Compared with controls, the EP cohort had more multiple births and complications associated with preterm birth including brain injury, necrotising enterocolitis, postnatal corticosteroids, and oxygen requirement at 36 weeks’ corrected gestational age, as expected (Table 1). The mid-parental height was lower in the EP group compared with controls (mean z-scores −0.37 vs −0.14, respectively).

**Table 1:** Participant characteristics.

	EP (n = 225)	Term controls (n = 252)
Antenatal corticosteroids, n (%)	160 (71.1)	1/249 (0.4)
Multiple birth, n (%)	73 (32.4)	6 (2.4)
Male, n (%)	113 (50.2)	121 (48.0)
Gestation at birth (completed weeks)	25.9 (1.1)	39.3 (1.3)
Birth weight (g)	891 (176)	3398 (440)
Birth weight z-score	0.07 (0.89)	0.12 (0.92)
Small for gestational age (<-2 SD), n (%)	5 (2.2)	1 (0.4)
Neonatal brain injury,^c^ n (%)	30 (13.3)	0 (0)
Oxygen at 36 weeks, n (%)	104 (46.2)	0 (0)
Postnatal corticosteroids,^d^ n (%)	91 (40.4)	0 (0)
Mid-parental height z-score	−0.37 (0.89) n = 162	−0.14 (0.90) n = 152
**Growth parameters**		
**2 years**	**n** **=** **215**	**n** **=** **228**
Corrected age at follow-up (years)	2.0 (0.2)	2.1 (0.1)
Weight z-score	−0.37 (1.29)	0.49 (0.90)
Height z-score	−0.55 (1.18)	0.26 (0.96)
BMI z-score	−0.08 (1.15)	0.44 (1.02)
Overweight,^a^ n (%)	13/211 (6.2)	17/224 (7.6)
**5 years**	**n** **=** **211**	**n** **=** **217**

Corrected age at follow-up (years)	5.0 (0.2)	5.1 (0.2)
Weight z-score	−0.56 (1.50)	0.30 (1.06)
Height z-score	−0.34 (1.22)	0.25 (0.93)
BMI z-score	−0.55 (1.44)	0.16 (1.15)
Overweight,^a^ n (%)	22/210 (10.5)	35/214 (16.4)
**8 years**	**n** **=** **209**	**n** **=** **213**
Corrected age at follow-up (years)	8.7 (0.3)	8.9 (0.4)
Weight z-score	−0.31 (1.50)	0.39 (1.05)
Height z-score	−0.30 (1.28)	0.27 (0.99)
BMI z-score	−0.11 (1.41)	0.35 (1.15)
Overweight,^a^ n (%)	31/207 (15.0)	40/212 (18.9)
**18 years**	**n** **=** **166**	**n** **=** **152**
Corrected age at follow-up (years)	17.9 (0.8)	18.1 (0.9)
Weight z-score	0.07 (1.53)	0.46 (1.09)
Height z-score	−0.47 (1.14)	0.26 (0.99)
BMI z-score	0.40 (1.43)	0.43 (1.08)
BMI (kg/m^2^)	23.2 (5.2)	22.9 (3.5)
Overweight,^a^ n (%)	46 (27.7)	31 (20.4)
**25 years**	**n** **=** **128**	**n** **=** **126**
Corrected age at follow-up (years)	25.1 (0.7)	25.3 (0.9)
Weight z-score	0.37 (1.77)	0.75 (1.19)
Height z-score	−0.52 (1.09)	0.28 (0.97)
BMI z-score	0.45 (1.73)	0.43 (1.19)
BMI (kg/m^2^)	25.7 (7.5)	24.6 (3.9)
Overweight,^b^ n (%)	50 (39.1)	51 (40.5)

Data are mean (SD) unless otherwise specified.

EP = extremely preterm; SD = standard deviation; BMI = body mass index.

a Overweight = calculated using UK 1990.

b Overweight = absolute BMI ≥25 at 25 years.

c Intraventricular hemorrhage grade III/IV or cystic periventricular leukomalacia.

d To treat or prevent bronchopulmonary dysplasia.

EP participants who were assessed at 25 years were more likely to have received antenatal corticosteroids than those who were not assessed. Other characteristics were similar. Controls who were assessed at 25 years were similar to those who were not (Supplementary Table S1).

### Comparison of growth between EP and controls (Table 1 and Fig. 1)

The proportions of those overweight in both groups increased more than 6-fold between 2 and 25 years. EP participants had lower z-weight, and z-height at all ages compared with controls. Although z-BMI was lower in the EP group compared with controls at younger ages, the differences were negligible from age 18 years.

**Fig. 1:**
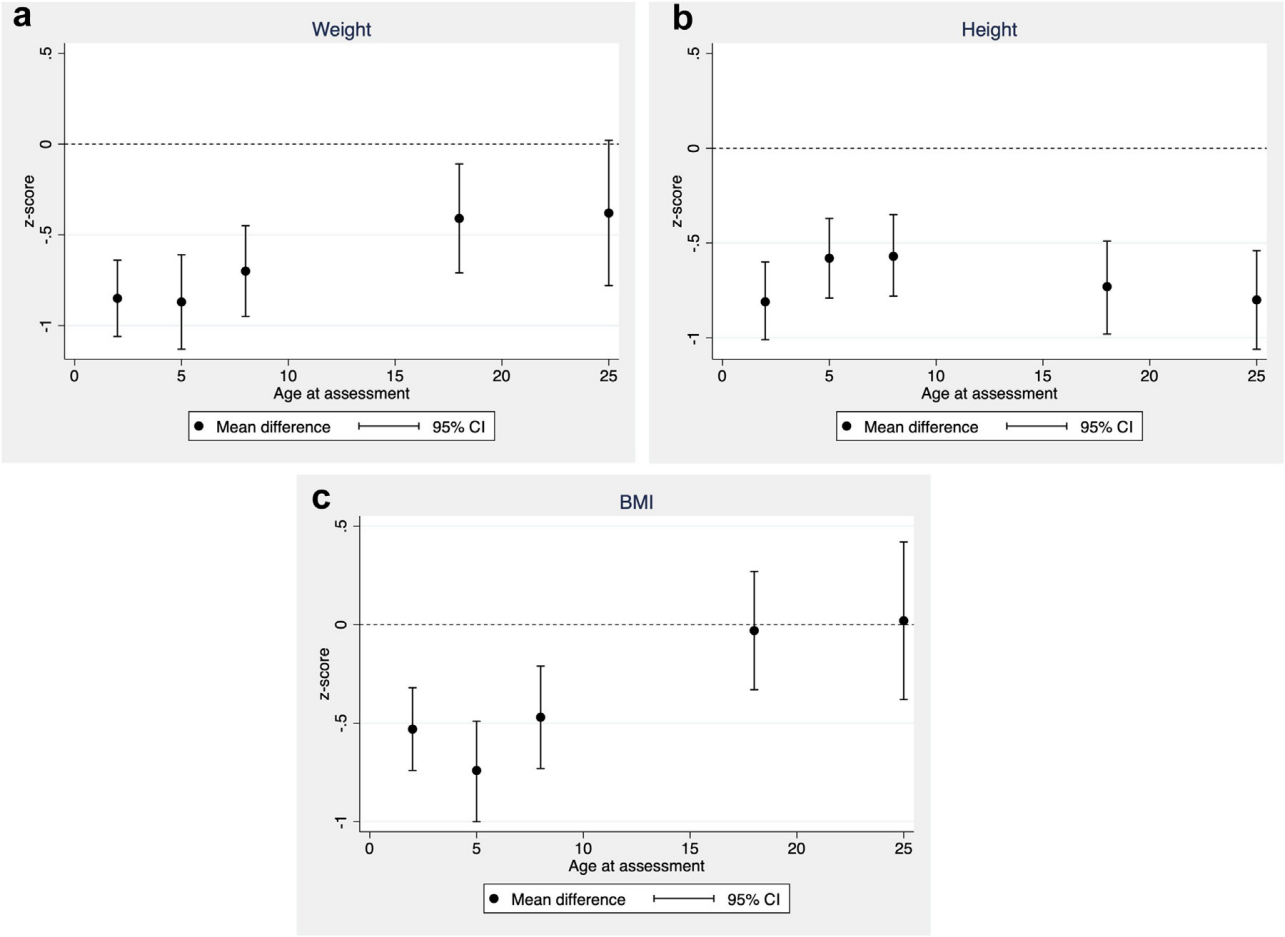
**Mean differences (95% confidence intervals) in body size z-scores between EP and controls at each age: (a) Weight; (b) Height; (c) BMI**.

### Growth trajectories between 2 and 25 years (Fig. 2)

#### Effect of EP birth and interaction between group and age

Z-weight increased with age in both groups, with the rate of increase with age greater in the EP group compared with controls (interaction p = 0.004).

**Fig. 2:**
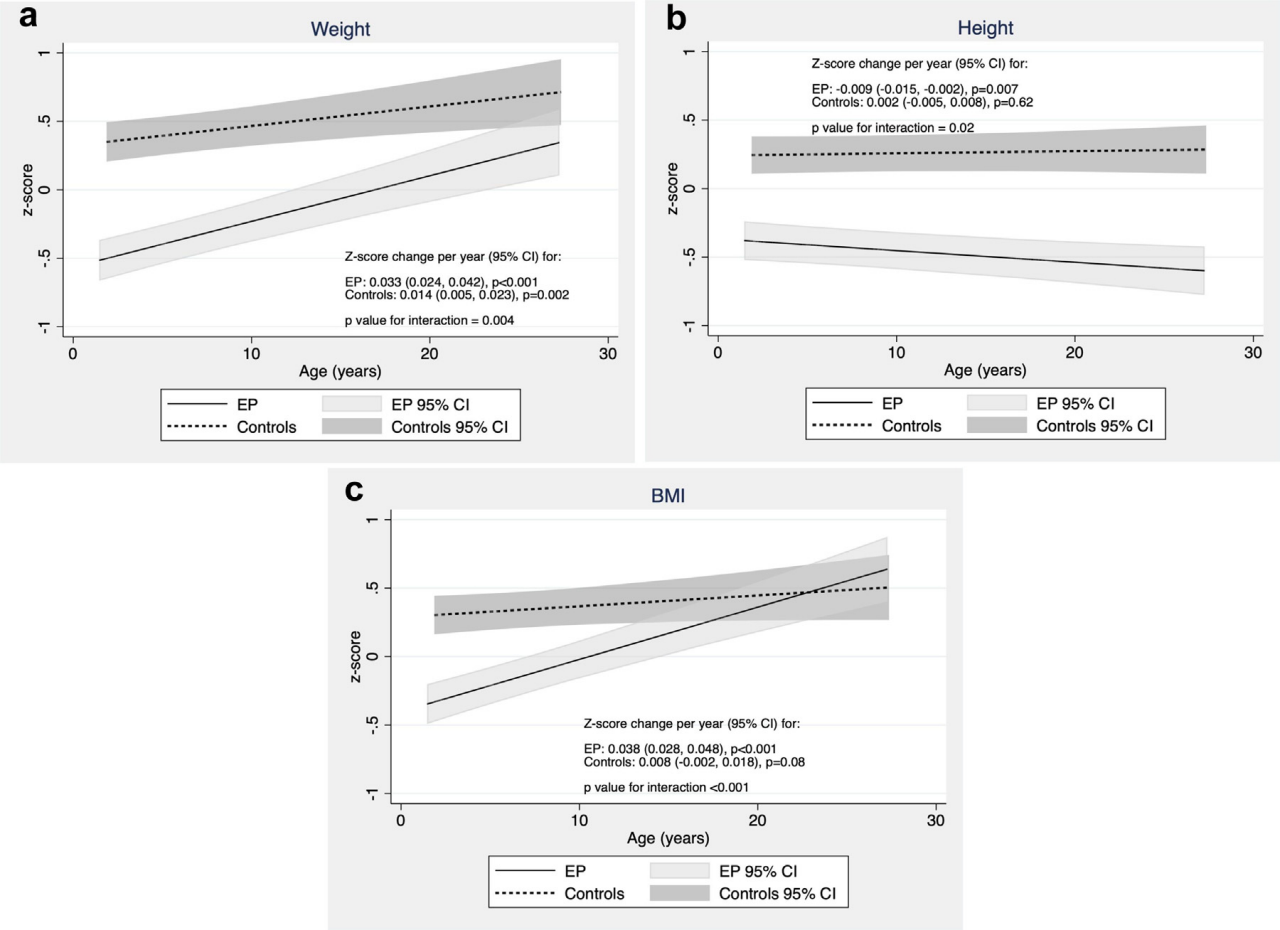
**Growth trajectories contrasted between EP and controls: (a) Weight; (b) Height; (c) BMI**.

Although z-height remained relatively constant with age, z-height decreased over time in the EP group but not controls (interaction p = 0.02).

Z-BMI increased with age in both groups, with a larger rate of increase in the EP group (interaction p < 0.001).

#### Effect of sex and interaction between sex and age

There was little evidence for differences in z-weight, z-height, or z-BMI between males and females (data not shown). Z-score changes with increasing age for all three growth parameters were similar between males and females (p-values for interactions >0.05).

### Associations with cardiometabolic health in the EP group

Group comparisons of the cardiometabolic variables have been reported previously,^17^^,^^20^ with the means and standard deviations for the EP group summarised in Table 2. Greater increases in z-BMI/year between 2 and 25 years were associated with changes in most variables that were consistent with less favourable cardiometabolic health at age 25 years, except for cholesterol and LDL where the evidence for associations was weak (Table 2). For skewed outcomes we have also estimated the difference in medians. These results are provided in Supplementary Table S2.

**Table 2:** Associations between rate of BMI z-score change with cardiometabolic health at age 25 in the EP group.

Variable (unit)	n	Mean (SD)	Coefficient (95% CI)^a^	p value
**Body composition**				
Visceral fat volume (cm^3^)	123	431.8 (254.1)	217.8 (160.9, 274.7)	<0.001
**Glucose tolerance and cholesterol profile**				
Fasting insulin (mU/L)	125	9.55 (7.24)	6.13 (3.82, 8.45)	<0.001
Fasting glucose (mmol/L)	125	4.78 (0.64)	0.24 (0.14, 0.35)	<0.001
HOMA–IR	125	2.06 (1.63)	1.43 (0.91, 1.95)	<0.001
Cholesterol (mmol/L)	125	4.73 (0.85)	0.13 (−0.08, 0.34)	0.22
HDL (mmol/L)	125	1.33 (0.36)	−0.21 (−0.28, −0.14)	<0.001
LDL (mmol/L)	123	2.88 (0.78)	0.19 (−0.04, 0.42)	0.10
Triglycerides (mmol/L)	125	1.17 (0.95)	0.45 (0.20, 0.71)	0.001
**Blood pressure**				
Systolic blood pressure 24 hr	115	129.7 (14.1)	8.9 (5.8, 12.0)	<0.001
Diastolic blood pressure 24 hr	115	72.7 (8.4)	4.6 (2.6, 6.6)	<0.001
**Exercise capacity**				
Six-minute walk maximum distance (m)	124	613.9 (89.2)	−53.3 (−74.7, −32.0)	<0.001
BEEP test maximum level	112	4.6 (2.3)	−1.2 (−1.7, −0.7)	<0.001

BMI = body mass index; EP = extremely preterm; HOMA-IR = homeostasis model assessment insulin resistance index; HDL = high-density lipoproteins; LDL = low-density lipoproteins; MAP = mean arterial pressure.

a Per 0.1 increase in z-score/year.

**Fig. 3:**
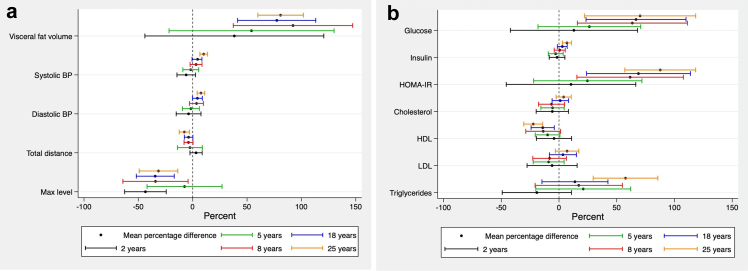
**Differences in mean cardiometabolic health outcomes at age 25 years between overweight and non-overweight EP-born individuals at each age, expressed as a percentage of the overall mean of the outcome. Positive values indicate larger outcome values for overweight individuals, compared with non-overweight individuals. (a) Physical variables, (b) Laboratory variables**.

The associations between being overweight with poorer cardiometabolic health in young adulthood strengthened progressively with age (Fig. 3). Although evidence for these associations was weak at younger ages, associations strengthened from around school age (i.e., 8 years) onwards. Specifically, being overweight from age 8 years was associated with higher visceral fat volume, higher fasting glucose, insulin and HOMA-IR. From age 18 years, being overweight was associated with lower HDL and higher systolic blood pressure. By age 25 years, evidence for associations between being overweight, with less favourable cardiometabolic health was present for all variables except for cholesterol and LDL.

## Discussion

Overall EP participants remained shorter and lighter than controls through childhood and into young adulthood. However, “catch-up” growth in the EP group was observed whereby the gap in weight and BMI between groups diminished with age, and by age 25 years, z-BMI in both groups was similar. Greater rates of BMI rise from infancy to young adulthood were associated with poorer cardiometabolic health in the EP group, as was being overweight or obese in childhood through young adulthood. The evidence for the latter was weak at younger ages but strengthened from mid-childhood (8 years). For both groups, the high proportion (40%) at 25 years who were overweight is concerning.

The gap in growth for weight, height and BMI between EP and controls has been reported previously, in cohorts selected by gestational age^2^^,^^3^ or by birthweight.^11^ The EPICure cohort in the United Kingdom recruited a cohort born ≤25 weeks’ gestation, and tracked their growth compared with controls up to age 19 years.^3^ They found similar trends in the trajectories of growth to the present study, with the greatest relative increases in weight and BMI from age 6–11 years, but not thereafter. In contrast, the present study showed “catch-up” weight and BMI persisted past late adolescence to young adulthood and by 25 years, the group differences were considerably attenuated. The catch-up gains in the EP group reported by EPICure were greater than those in our study; mean (SD) per year for z-weight 0.06 (0.05, 0.07) and z-BMI 0.08 (0.07, 0.10) compared with z-weight 0.03 (0.02, 0.04) and z-BMI 0.04 (0.03, 0.05) respectively.^3^ This could in part be explained by the cohort characteristics, where we included more mature infants (26 and 27 weeks).

The height trajectories reported in our study are unique. Although the disparity in height between EP and controls persisted to adulthood, there was evidence that height trajectories were decreasing with age in the EP group, but not controls. The reason for this observation is unclear. Reductions in height have been described in general populations from the age of 40 years, often in conjunction with worsening general health and hypotheses that changes in muscle mass and bone density may in part be contributory factors, especially in females.^21^ We did not find evidence of differences between males and females for changes in height trajectories with age. Although we have demonstrated group differences in bone health in these cohorts consistent with bone demineralisation in the EP group compared with controls,^22^ one would not have expected this to explain the decrease in height trajectories observed in young adulthood. However, further follow-up to track growth and bone health in these cohorts into mid-adulthood and older is important to determine if these trends continue.

Van der Pol et al.^11^ conducted a systematic review of studies reporting growth patterns in preterm cohorts selected by birth weight. There were 17 studies of cohorts born extremely low birth weight (<1000 g) with follow up to age 34 years. Weight and height were consistently lower in the ELBW group compared with normal birth weight controls. Results were inconclusive for BMI. Most studies in that systematic review reported “catch-up” weight and height, defined as a positive increase of the z-score of >0.67 over a prespecified period of time, or reaching growth parameters more than −2 SD scores for age, that continued until adulthood. It is difficult to directly compare the findings of the systematic review to the present study due to the potential bias of cohorts selected by birth weight, and inclusion of cohorts born before the 1990s which may not include the most immature infants due to lower survival before 1990.

The present study identified several parameters of growth that were associated with poorer cardiometabolic health in young adulthood. Greater rates of rise in BMI between 2 and 25 years were associated with less optimal body composition, poorer glucose tolerance and cholesterol profile, higher blood pressure and poorer exercise capacity. These observations provide further evidence that promoting “excessive” catch-up growth in EP children may be associated with later cardiometabolic morbidity. In a national cohort in Finland, Barker et al. identified that catch up BMI growth between age 2 and 11 years was associated with later coronary events and insulin resistance,^23^ findings that have been replicated in subsequent cohorts.^24^ Given that EP cohorts have poorer adult cardiometabolic health than term controls,^20^^,^^25^ it is important to balance the risk-benefit of maximising growth in EP children. More research is needed to determine optimal “catch-up” growth targets that clinicians should aim for when caring for EP populations.

Although the association between overweight in childhood with metabolic syndrome^26^ or cardiovascular disease in later life has been described,^23^^,^^24^ the strength of the associations at different ages is less clear, especially for EP populations. Our findings that associations strengthened with increasing age is unique. Thus, there may be a window of opportunity to intervene if EP children are identified as being overweight at earlier ages to alter the trajectory of growth and risk of later cardiometabolic disease. Dietary and lifestyle interventions for a healthy weight need to be designed to suit EP populations, who may have other physical and cognitive challenges that render population-based weight reduction interventions unsuitable.

The current study has several strengths, including the geographic nature of the EP cohort, recruited at birth and prospectively followed up to 25 years, and the inclusion of contemporaneous term-born controls with which to compare outcomes. Unlike previous cohorts selected by birth weight, we selected by gestational age to overcome the potential bias of growth restriction in more mature individuals. We had repeated growth measurements spanning infancy, through childhood into young adulthood, and a broad range of cardiometabolic health variables in adulthood to enable a broad understanding of the relationships with growth. Limitations include the attrition rates which are common with longitudinal cohort studies of similar duration and may lead to selection bias. We were able to compare the clinical characteristics between those who were assessed and those who were not, and by using mixed effects model and calculating individual slopes in our analysis we were able to use as much of the available data as possible. We did not assess for pubertal stages in the cohort, which may affect growth rates. However, we have previously reported that pubertal stages at 14 years of age were similar between an extremely preterm cohort compared with controls in another cohort from the same geographical region.^27^ We did not have the resources to study the biochemical basis for some of our findings. Our analysis was descriptive in nature, i.e., we did not aim to estimate causal effects (which require consideration of confounding). The associations observed in this study should not be interpreted as causal effects.

### Conclusions

Poor growth in EP children is well-recognised, and there are multiple efforts focussed on maximising nutrition to enhance growth. However, with evidence that growth trajectories in EP cohorts may be suboptimal compared with term controls, and the association with poorer cardiometabolic health adulthood with catch-up growth in weight and BMI, there needs to be more research into the optimal growth trajectories of the EP group. Further it is critical that follow-up of EP cohorts be strongly supported to understand the trajectories into later life, and the associations with cardiometabolic health that is the commonest morbidity facing high-income countries.

## Contributors

Drs Cheong, Olsen, Konstan, and Doyle–conception and design of the study, data analysis and interpretation, drafting and revising the article, and approval of the final manuscript as submitted.

Dr Mainzer – data analysis and interpretation, revising the article, and approval of the final manuscript as submitted.

Drs Hickey, Spittle, Wark, Cheung, Garlamd, Duff, Clark, and Stevens – data collection, revising the article, and approval of the final manuscript as submitted.

## Data sharing statement

The data from this study is not freely available but may be available as part of collaborative projects upon agreement with data custodians.

## Declaration of interests

The authors have no conflicts of interest relevant to this article to disclose.
