# Supplementary material for: Growth from infancy to adulthood and associations with cardiometabolic health in individuals born extremely preterm
Source: Lancet Reg Health West Pac. 2023 Feb 27;34:100717. doi: 10.1016/j.lanwpc.2023.100717 (PMC10240366; doi:10.1016/j.lanwpc.2023.100717)
Supplement: Supplemental Tables S1 and S2 [file mmc1.docx]

**Supplementary Table 1: Comparisons between participants who were assessed at 25 years and those who were not^$^**

|  | **EP** | | | **Controls** | | |
| --- | --- | --- | --- | --- | --- | --- |
|  | **Seen**  **n=128** | **Not seen**  **n=97** | **Mean diff or Odds ratio**  **(95% CI)^**  **p value** | **Seen**  **n=127** | **Not seen**  **n=125** | **Mean diff or Odds ratio**  **(95% CI)^**  **p value** |
| Antenatal corticosteroids, n (%) | 98 (76.6) | 62 (63.9) | 1.84  (1.03, 3.30)  p=0.04 | 0 (0) | 1 (0.8) | - |
| Multiple birth, n (%) | 45 (35.2) | 28 (28.9) | 1.34  (0.76, 2.36)  p=0.32 | 2 (1.6) | 4 (3.2) | 0.48  (0.09, 2.69)  p=0.41 |
| Male, n (%) | 62 (48.4) | 51 (52.6) | 0.85  (0.50,1.44)  p=0.54 | 54 (42.5) | 67 (53.6) | 0.64  (0.39, 1.05)  p=0.08 |
| Gestation at birth (completed weeks) | 25.8 (1.1) | 26.0 (1.1) | -0.2  (-0.5, 0.1)  p=0.09 | 39.3 (1.3) | 39.3 (1.2) | -0.1  (-0.3, 0.3)  p=0.86 |
| Birth weight (g) | 878 (171) | 908 (182) | -30  (-76, 17)  p=0.21 | 3387 (461) | 3408 (420) | -21  (-130, 88)  p=0.71 |
| Birth weight z-score | 0.06 (0.86) | 0.08 (0.92) | -0.01  (-0.25, 0.22)  p=0.92 | 0.12 (0.94) | 0.11 (0.91) | 0.01  (-0.23, 0.23)  p=0.98 |
| Small for gestational age (<-2 SD), n (%) | 4 (3.1) | 1 (1.0) | 3.10  (0.34, 28.16)  P=0.32 | 1 (0.8) | 0 (0) | - |

^$^Of survivors to age 2 years; ^Comparisons from linear or logistic regression as appropriate.

EP = extremely preterm (22-27 weeks’ gestation); SD = standard deviation; CI = confidence interval.

**Supplementary Table 2: Associations between rate of BMI z-score change with cardiometabolic health at age 25 in the EP group for variables with a skewed distribution**

| **Variable (unit)** | **n** | **Median**  **(IQR)** | **Difference of medians (95% CI)#** | **p value** |
| --- | --- | --- | --- | --- |
| Visceral fat volume (cm^3^) | 123 | 349.9  (264.2, 543.1) | 168.6  (107.9, 229.3) | <0.001 |
| Fasting insulin (mU/L) | 125 | 6.9  (4.9, 11.5) | 3.73  (2.34, 5.12) | <0.001 |
| HOMA - IR | 125 | 1.50  (1.05, 2.56) | 0.88  (0.58, 1.18) | <0.001 |
| Triglycerides (mmol/L) | 125 | 0.9  (0.7, 1.3) | 0.31  (0.18, 0.43) | <0.001 |

# per 0.1 increase in z-score/year

IQR = interquartile range, HOMA-IR = homeostasis model assessment insulin resistance index
